# miRNA-27a Transcription Activated by c-Fos Regulates Myocardial Ischemia-Reperfusion Injury by Targeting ATAD3a

**DOI:** 10.1155/2021/2514947

**Published:** 2021-08-08

**Authors:** Yandong Bao, Ying Qiao, Hang Yu, Zeying Zhang, Huimin Yang, Xin Xin, Yuqiong Chen, Yuxuan Guo, Nan Wu, Dalin Jia

**Affiliations:** ^1^Department of Cardiology, The First Affiliated Hospital of China Medical University, Liaoning, China; ^2^The Central Laboratory, The First Affiliated Hospital of China Medical University, Liaoning, China; ^3^Department of Oromaxillofacial-Head and Neck Surgery, Department of Oral and Maxillofacial Surgery, School of Stomatology, China Medical University, China

## Abstract

MicroRNA-27a (miR-27a) has been implicated in myocardial ischemia-reperfusion injury (MIRI), but the underlying mechanism is not well understood. This study is aimed at determining the role of miR-27a in MIRI and at investigating upstream molecules that regulate miR-27a expression and its downstream target genes. miR-27a expression was significantly upregulated in myocardia exposed to ischemia/reperfusion (I/R) and cardiomyocytes exposed to hypoxia/reoxygenation (H/R). c-Fos could regulate miR-27a expression by binding to its promoter region. Moreover, overexpression of miR-27a led to a decrease in cell viability, an increase in LDH and CK-MB secretion, and an increase in apoptosis rates. In contrast, suppression of miR-27a expression resulted in the opposite effects. ATPase family AAA-domain-containing protein 3A (ATAD3a) was identified as a target of miR-27a. miR-27a regulated the translocation of apoptosis-inducing factor (AIF) from the mitochondria to the nucleus and H/R-induced apoptosis via the regulation of ATAD3a. It was found that inhibiting miR-27a in vivo by injecting a miR-27a sponge could ameliorate MIRI in an isolated rat heart model. In conclusion, our study demonstrated that c-Fos functions as an upstream regulator of miR-27a and that miR-27a regulates the translocation of AIF from the mitochondria to the nucleus by targeting ATAD3a, thereby contributing to MIRI. These findings provide new insight into the role of the c-Fos/miR-27a/ATAD3a axis in MIRI.

## 1. Introduction

With the dramatic changes in lifestyle and diet that have occurred in modern society, coronary artery disease (CAD) has gradually become one of the major diseases that seriously threaten the lives and health of people worldwide [1]. Severe stenosis or acute occlusion of the coronary arteries can cause myocardial ischemia and even myocardial necrosis. A common approach for treating patients with acute myocardial infarction (AMI) is reconstituting the myocardial blood supply as quickly as possible through the implementation of myocardial reperfusion therapy [2, 3], which includes percutaneous coronary intervention, coronary artery bypass grafting, and thrombolytic therapy. However, during the implementation of myocardial reperfusion therapy, the rapid recovery of the blood supply to the ischemic myocardium does not ameliorate myocardial damage and causes extramyocardial insult, which is termed myocardial ischemia-reperfusion injury (MIRI) [4]. The occurrence of MIRI is difficult to predict in advance, and once it occurs, it greatly reduces the clinical benefit of myocardial reperfusion therapy [5]. Therefore, it is of great significance to explore the mechanism underlying MIRI and to discover new therapeutic targets for MIRI.

MicroRNAs (miRNAs), short noncoding RNA molecules approximately 21 to 24 nucleotides in length, generally play roles in RNA silencing and regulate gene expression at the posttranscriptional level [6]. miR-27a has been widely reported to play key roles in the initiation and progression of cancer [7, 8], the occurrence of pulmonary and hepatic fibrosis [9, 10], and the development of arthritis [11]. Notably, miR-27a expression was significantly increased in mouse hearts subjected to ischemia-reperfusion, and downregulation of miR-27a expression mediates the protective effect of high thoracic epidural block against MIRI in mice [12]. However, the upstream molecule that regulates miR-27a expression and its downstream target genes have not been determined.

c-Fos, a member of the Fos family of transcription factors (including c-Fos, FosB, Fra-1, and Fra-2) [13], promotes the formation of the AP-1 transcription factor complex by dimerizing with the c-Jun protein, thereby translating extracellular signals into alterations in gene expression by binding to the promoters of target genes [14]. The level of c-Fos is notably changed under different stress conditions, such as heat stress [15], radiation [16], and ischemia [17]. Accumulating evidence has indicated that c-Fos expression is strongly induced during MIRI [18, 19]. More importantly, c-Fos was reported to increase miRNA expression by binding to miRNA promoters [20, 21]. Moreover, a c-Fos-specific binding site was predicted to exist in the putative promoter region of miR-27a by the PROMO database. Therefore, whether c-Fos regulates miR-27a expression was examined in the present study.

ATPase family AAA-domain-containing protein 3A (ATAD3a), a nuclear DNA-encoded mitochondrial membrane protein [22], has been reported to function in apoptosis [23, 24], mitochondrial dynamics [25], mitophagy [26], mitochondrial DNA replication [27, 28], and cholesterol metabolism [28, 29]. As predicted with TargetScan, miR-27a may specifically bind to the 3′-UTR of ATAD3a. Therefore, whether miR-27a regulates MIRI by targeting ATAD3a was determined in this study.

In the present study, we found that c-Fos activated the transcription of miR-27a and that miR-27a further regulated the ischemia-reperfusion-induced apoptosis of cardiomyocytes by modulating the translocation of apoptosis-inducing factor (AIF) from the mitochondria to the nucleus by targeting ATAD3a. These data suggest that the c-Fos/miR-27a/ATAD3a axis plays a key role in MIRI.

## 2. Materials and Methods

### 2.1. miRNA Array Analysis

To explore the differential expression of miRNAs in rats exposed to myocardial ischemia-reperfusion, we searched the GEO database and found the GSE74951 dataset that was contributed by Feng et al. [30]. The miRNA expression profile based on the GPL21136 Multiplex Circulating miRNA Assay was downloaded. We used the “limma R” language package to screen DE-miRNAs between ischemia-reperfusion-treated heart samples and normal heart samples. The cutoff criteria were set to *p* < 0.05 and ∣log2 multiple change (FC) | >1.

### 2.2. Animals and Animal Models

Thirty healthy male Wistar rats (body weight 250 ± 20 g) were purchased from Sibefu Biotechnology Co., Ltd. (Sibefu, Beijing, China). All the rats were used and handled in accordance with the Guidelines for Care and Use of Laboratory Animals provided by the National Institute of Health. The use of animals was approved by the Animal Ethics Committee of China Medical University.

The rats were injected with heparin (1500 IU/kg) before surgery to prevent blood clotting in the coronary arteries, and then, the rats were anesthetized by intraperitoneal injection with isopentobarbital (50 mg/kg). The fully anesthetized rats were subjected to thoracotomy. After the aorta was cut, the heart was isolated and immediately immersed in cold heparinized and oxygenated Krebs-Henseleit (KH) solution. The isolated heart was fixed on the Langendorff device and perfused with KH solution at a constant pressure of 75 mmHg and 37°C. MIRI was induced in the isolated rat hearts by interrupting perfusion for 30 min, which was followed by reperfusion for 90 min as previously described [31].

### 2.3. Cells and Cell Models

H9c2 cells purchased from the National Collection of Authenticated Cell Cultures were cultured in DMEM containing 10% fetal bovine serum, 100 U/ml penicillin, and 100 *μ*g/ml streptomycin. The culture conditions in the incubator were 37°C and 5% CO_2_.

An in vitro MIRI model was established as previously described [32]. H9c2 cells were collected in the logarithmic phase of growth, and the normal medium was replaced with Earle's solution without glucose or serum. The cells were placed in a three-gas incubator (94% N_2_, 5% CO_2_, and 1% O_2_; 37°C) and subjected to hypoxia for 8 h. Subsequently, Earle's solution was replaced with normal medium, and the cells were reoxygenated in a standard incubator (5% CO_2_, 37°C) for 3 h.

### 2.4. RT-qPCR

Total RNA was extracted from myocardial tissues and H9c2 cells using TRIzol™ Reagent (Invitrogen, Carlsbad, CA) according to the manufacturer's instructions. The concentration of RNA was measured using a NanoDrop2000 (Thermo Fisher Scientific, Wilmington, DE). The PrimeScript™ RT reagent Kit (Takara, Japan) and Mir-X miRNA First-Strand Synthesis Kit (Clontech, Japan) were used to reverse transcribe miRNA and mRNA, respectively, into cDNA. Real-time quantitative PCR was performed using TB Green® Premix Ex Taq™ II (Takara, Japan) according to the manufacturer's instructions, and the real-time quantitative PCR was carried out with the QuantStudio real-time fluorescent quantitative PCR system (Thermo Fisher Scientific, Wilmington, DE). The primers used were provided by Sangon Biotech (Shanghai, China): miR-27a forward (5′-AGGGCTTAGCTGCTTGTGAGC-3′), miR-27a reverse (5′-CGGCAGAGTCCTTACCCACAA-3′, U6 reverse primer (5′-TGGAACGCTTCACGAATTTGCG-3′), U6 forward primer (5′-GGAACGATACAGAGAAGATTAGC-3 ′), ATAD3a forward (5′-GATGACGATATGCGGCTGGTACAC-3′), ATAD3a reverse (5′-GATGACGATATGCGGCTGGTACAC-3′), GAPDH forward (5′-CTGGAAAGCTGTGGCGTGAT-3′), and GAPDH reverse (5′-GCGGCATGTCAGATCCACAA-3′).

### 2.5. Cell Transfection

Short miRNA sequences, small interfering RNAs specific for c-Fos and ATAD3a, c-Fos, and ATAD3a plasmids, and matched negative controls or empty vectors were designed and synthesized by GenePharma (GenePharma Co., Ltd., Shanghai, China). These reagents were transfected into cells using INVI DNA/RNA Transfection Reagent™ (Ivigentech, America) according to the manufacturer's instructions. Subsequent cell experiments were performed 24 h or 48 h after transfection.

### 2.6. Cell Counting Kit-8 (CCK-8) Assay

H9c2 cells were seeded in 96-well plates (3000 cells per well), and cell viability was assessed using a CCK-8 assay (APExBIO, Houston, USA) according to the manufacturer's protocol.

### 2.7. Determination of Myocardial Enzyme Levels

The levels of lactate dehydrogenase (LDH) and creatine kinase-MB (CK-MB) in the culture medium were measured using the Lactate Dehydrogenase Release Kit (Jiancheng Bioengineering Institute, Nanjing, China) and the Creatine Kinase-MB Isoenzyme Assay Kit (Jiancheng Bioengineering Institute, Nanjing, China) according to the manufacturer's instructions.

### 2.8. Flow Cytometry

Cell apoptosis in vitro was detected using the Annexin V FITC Apoptosis Detection Kit (Dojindo, Japan) following the manufacturer's instructions.

### 2.9. Gene Therapy In Vivo

As adeno-associated virus serotype 9 (AAV9) is superior to other serotypes for global cardiac gene transfer [33], in this study, inhibition of miR-27a in vivo was achieved by injecting AAV9-miR-27a-sponge (HanBio, China) through the rat tail vein, and AAV9-NC was injected as a negative control. miR-27a expression was detected by RT-qPCR three weeks after the injection to assess the suppression rate.

### 2.10. Transmission Electron Microscopy

The left ventricle was cut into a 1 × 1 × 1 mm cube, fixed with 2.5% glutaraldehyde, and then cut into ultrathin sections. The sections were observed under a transmission electron microscope (JEM-1200EX, JEOL), and alterations in the myocardial submicroscopic structure were observed and photographed.

### 2.11. HE Staining

The left ventricle was fixed with 4% paraformaldehyde, dehydrated by gradient alcohol, embedded in paraffin, and cut into 4 *μ*m-thick sections. The sections were stained with hematoxylin and eosin (Beyotime Biotechnology, China) according to the manufacturer's recommendations, followed by observation under an optical microscope (Olympus BX51, Olympus).

### 2.12. 2,3,5-Triphenyltetrazolium Chloride (TTC) Staining

The myocardial infarction area was determined using TTC staining. The perfused heart was harvested at the end of reperfusion and frozen at -20°C. The frozen heart was cut into 1 mm-thick sections, incubated in 1% TTC solution (Solarbio, China) at 37°C for 30 min in the dark and then fixed with 4% paraformaldehyde (Solarbio, China) overnight. The stained sections were photographed using a digital camera.

### 2.13. Terminal Deoxynucleotidyl Transferase-Mediated dUTP-Biotin Nick End Labeling (TUNEL) Assay

A TUNEL staining kit (Roche Diagnostics, Germany) was used to detect apoptosis in the myocardium, and apoptotic cells were observed under an inverted fluorescence microscope (Nikon Eclipse TE2000-U, Japan).

### 2.14. Chromatin Immunoprecipitation (ChIP) Assay

ChIP was conducted using a ChIP Assay Kit (cat# 17-295, EMD Millipore, Billerica, MA, USA). After fixation with 1% formaldehyde for 10 min, the cells were subjected to decrosslinking with 0.125 M glycine for 5 min, washed with PBS, and lysed for 1 h on ice. The cell lysates were sonicated to generate chromatin fragments approximately 500 to 800 bp in length that were assessed by agarose gel electrophoresis. Following preclearing with Protein-A agarose, the samples were incubated with 5 *μ*g of specific antibodies with rotation overnight at 4°C. Then, the immune complexes were precipitated with Protein-A agarose beads and sheared salmon sperm DNA, and the DNA fragments were purified using a QIAquick Spin Kit (Qiagen). The promoter segments containing a c-Fos binding site were amplified using PCR technology.

### 2.15. Luciferase Reporter Gene Assay

The PROMO database (http://alggen.lsi.upc.es/cgi-bin/promo_v3/promo/promoinit.cgi?dirDB=TF_8.3/) was used to predict the c-Fos-specific binding site in the promoter region of miR-27a. To determine the specific binding of c-Fos to the miR-27a promoter, the wild-type full-length promoter of miR-27a and the corresponding sequence with mutated c-Fos binding sites were cloned into pGL3 luciferase reporter vectors (GenePharma Co., Ltd., Shanghai, China), cotransfected with pcDNA3.1 vector or pcDNA3.1-c-Fos, and detected with the Dual-Glo Luciferase Assay System (Promega) according to the manufacturer's recommendations.

TargetScan version 7.2 (http://www.targetscan.org/) was used to predict the specific binding of miR-27a to the 3′-UTR of ATAD3a. Sequences of the ATAD3a 3′-UTR containing the wild-type or mutant miR-27a binding site were amplified by PCR and cloned into pmirGLO luciferase reporter vectors (GenePharma Co., Ltd., Shanghai, China) and cotransfected with mimic NC or miR-27a mimic. Then, the luciferase activities were measured as described above.

### 2.16. Western Blotting Analysis

RIPA lysis buffer (Beyotime Biotechnology, China) was used to extract the proteins from myocardial tissues and H9c2 cells, and the protein concentration was determined with the BCA protein concentration determination kit (Beyotime Biotechnology, China). After quantification, the protein samples were separated by SDS-PAGE and transferred to PVDF membranes. The membranes were blocked with 5% BSA for 2 h. After incubation with primary antibodies, including ATAD3a (1 : 2000, ProteinTech, Rosemont, USA), AIF (1 : 8000, ProteinTech, Rosemont, USA), *β*-actin (1 : 5000, 20536-1-AP, ProteinTech, Rosemont, USA), histone H3 (1 : 6000, 17168-1-AP, ProteinTech, Rosemont, USA), and COXIV (1 : 10000, Proteintech, Rosemont, USA) antibodies, overnight at 4°C, the membranes were incubated with HRP-labeled secondary antibodies for 1 h at room temperature. All the antibodies were purchased from Abcam (UK) and diluted according to the manufacturer's instructions. The Western blots were developed using the Pierce™ ECL Western blotting substrate (Thermo Scientific™, USA).

## 3. Statistical Analysis

All the data are expressed as the mean ± SD, and statistical analysis was performed with the GraphPad Prism version 8.0 software (San Diego, California, USA). To compare the differences between two groups, Student's *t*-test was used. When comparing differences among more than two groups, one-way ANOVA was performed, followed by multiple comparison analysis using Fisher's least significant difference test. *p* < 0.05 was considered statistically significant.

## 4. Results and Discussion

### 4.1. miR-27a Expression Was Induced by MIRI

**Figure 1:**
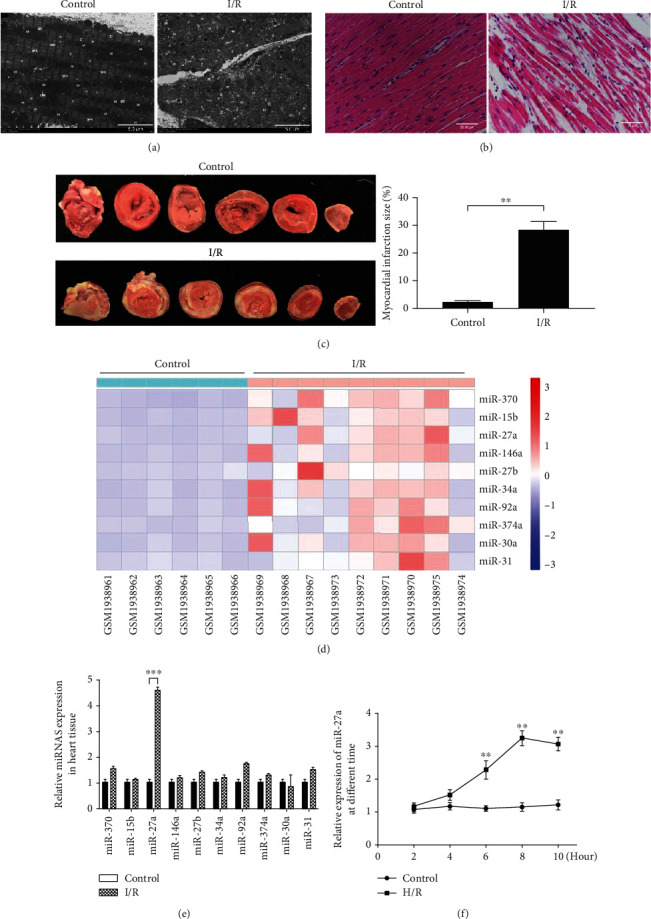
miR-27a expression was induced by myocardial ischemia/reperfusion injury (MIRI). Ischemia-reperfusion was induced by 30 min of ischemia followed by 90 min of reperfusion in an isolated rat heart model. Myocardial structure damage was detected by (a) transmission electron microscopy and (b) HE staining, *n* = 3. (c) Myocardial infarct size was measured by TTC staining, *n* = 6, ^∗∗^*p* < 0.01 vs. the control. (d) Heat maps of the top ten upregulated miRNAs in myocardia subjected to I/R, *n* = 6 in the control group, *n* = 9 in I/R group. (e) MicroRNA expression in myocardia subjected to I/R was confirmed by RT-qPCR, *n* = 10, ^∗∗∗^*p* < 0.001 vs. the control. (f) miR-27a expression in H9c2 cells subjected to hypoxia for different times was examined by RT-qPCR. The data were obtained from four independent replicate experiments. ^∗∗^*p* < 0.01 vs. the control.

As shown in Figures 1(a)–1(c), isolated rat hearts that were subjected to I/R displayed mitochondrial swelling, cristae rupture, myofibrillar vacuolation, interstitial edema, and nuclear fragmentation, as well as large amounts of inflammatory cell infiltration and large areas of myocardial necrosis, demonstrating that the MIRI model was successfully established. The top 10 upregulated miRNAs in the myocardia subjected to I/R were selected by analysis of GEO data (Figure 1(d)). Verification of the expression of these miRNAs by qRT-PCR revealed that miR-27a expression was significantly upregulated (almost 4.5-fold) in the I/R group compared with the control group (Figure 1(e)). In addition, miR-27a expression in H9c2 cells exposed to sustained hypoxia for different times was also examined. The level of miR-27a peaked after 8 h of hypoxia, followed by 3 h of reoxygenation (Figure 1(f)). Altogether, these results suggested that miR-27a was induced by I/R.

### 4.2. c-Fos Regulated miR-27a Expression

**Figure 2:**
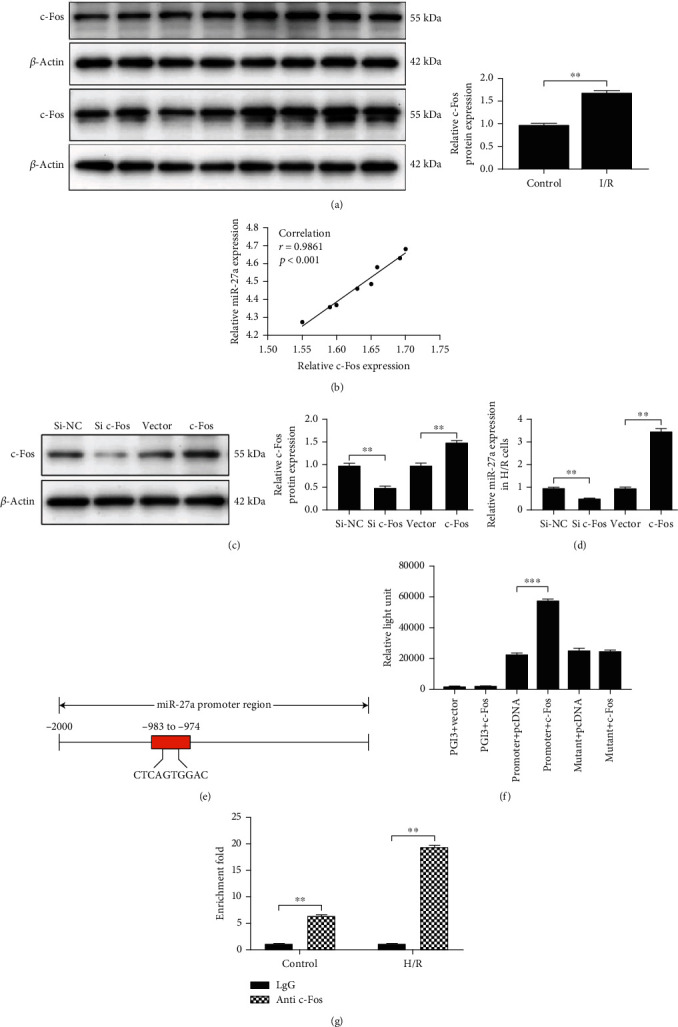
c-Fos regulated miR-27a expression. (a) c-Fos expression in myocardia subjected to ischemia/reperfusion (I/R) was analyzed by Western blotting, *n* = 8, ^∗∗^*p* < 0.01 vs. the control. (b) c-Fos expression was positively correlated with miR-27a expression in myocardia subjected to I/R. H9c2 cells were transfected with a small interfering RNA (siRNA) specific for c-Fos, c-Fos plasmid, and matched negative controls or empty vectors. (c) c-Fos expression was analyzed by Western blotting. (d) miR-27a expression was detected by RT-qPCR. (e) A c-Fos-specific binding site was predicted in the putative promoter region (-983 to -974 region) of miR-27a. (f) The relative luciferase activity was detected after cotransfecting pGL3 luciferase reporter vectors containing the full-length miR-27a promoter or the corresponding promoter sequence with a mutant binding site with the c-Fos plasmid and empty vector. (g) Hypoxia/reoxygenation (H/R) enhances the enrichment of c-Fos on the miR-27a promoter. H9c2 cells were subjected to H/R. Twenty-four hours after treatment, ChIP-qPCR was performed. All data were obtained from four independent replicate experiments. ^∗∗^*p* < 0.01; ^∗∗∗^*p* < 0.001.

Considering that miRNA expression can be regulated by transcription factors, whether miR-27a is transcriptionally activated by transcription factors was investigated. c-Fos is a key transcription factor that contributes to MIRI. As shown in Figure 2(a), c-Fos protein expression was upregulated in myocardia subjected to I/R, and the c-Fos level was positively correlated with miR-27a expression in these tissues (Figure 2(b)). Furthermore, inhibition of c-Fos expression using siRNA led to reduced miR-27a expression. Moreover, overexpression of c-Fos upregulated miR-27a expression (Figures 2(c) and 2(d)). Therefore, we focused on c-Fos as a putative upstream regulator of miR-27a expression.

As predicted by the PROMO database, a c-Fos-specific binding site existed in the putative promoter region (-983 to -974 region) of miR-27a (Figure 2(e)). The results of luciferase reporter assays indicated that cotransfection of a vector carrying the putative promoter with the pcDNA vector expressing c-Fos led to a remarkable increase in the relative luciferase activity compared with cotransfection of a vector carrying the putative promoter with the pcDNA empty vector. In contrast, there was no significant change in the relative luciferase activity after the cotransfection of a vector carrying the promoter with a mutated c-Fos binding site (Figure 2(f)). In addition, the ChIP-qPCR assay results further demonstrated that c-Fos could bind to the putative promoter region of miR-27a, and H/R treatment enhanced the enrichment of c-Fos on the putative promoter region of miR-27a (Figure 2(g)). Altogether, these results suggested that c-Fos regulates miR-27a expression.

### 4.3. miR-27a Regulated H/R-Induced Myocardial Injury In Vitro

**Figure 3:**
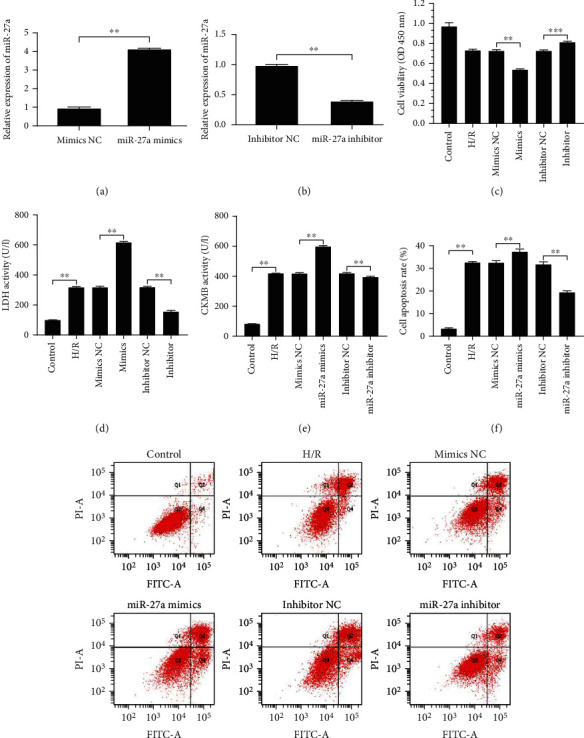
miR-27a regulated hypoxia/reoxygenation- (H/R-) induced myocardial injury in vitro. H9c2 cells were transfected with the miR-27a mimic, miR-27a inhibitor, mimics negative control (NC), and inhibitor NC, followed by 8 h of hypoxia and 3 h of reoxygenation. (a, b) miR-27 expression in H9c2 cells was detected by RT-qPCR after transfection with the miR-27a mimic or miR-27a inhibitor. (c) Analysis of cell viability by CCK-8 assay. (d, e) Measurement of LDH and CK-MB activity in the culture medium. (f) Cell apoptosis was detected by flow cytometry. All data were obtained from four independent replicate experiments. ^∗∗^*p* < 0.01; ^∗∗∗^*p* < 0.001.

To determine the role of miR-27a in H/R-induced injury, overexpression and suppression of miR-27a were achieved by transfecting cardiomyocytes with the miR-27a mimic and miR-27a inhibitor (Figures 3(a) and 3(b)), respectively. The results showed that overexpression of miR-27a led to a decrease in cell viability (Figure 3(c)), an increase in LDH (Figure 3(d)) and CK-MB (Figure 3(e)) secretion, and an increase in apoptosis (Figure 3(f)) in the cardiomyocytes subjected to H/R. In contrast, suppression of miR-27a led to the opposite effects. Altogether, these results suggested that miR-27a could regulate MIRI in vitro.

### 4.4. ATAD3a Was a Target of miR-27a

**Figure 4:**
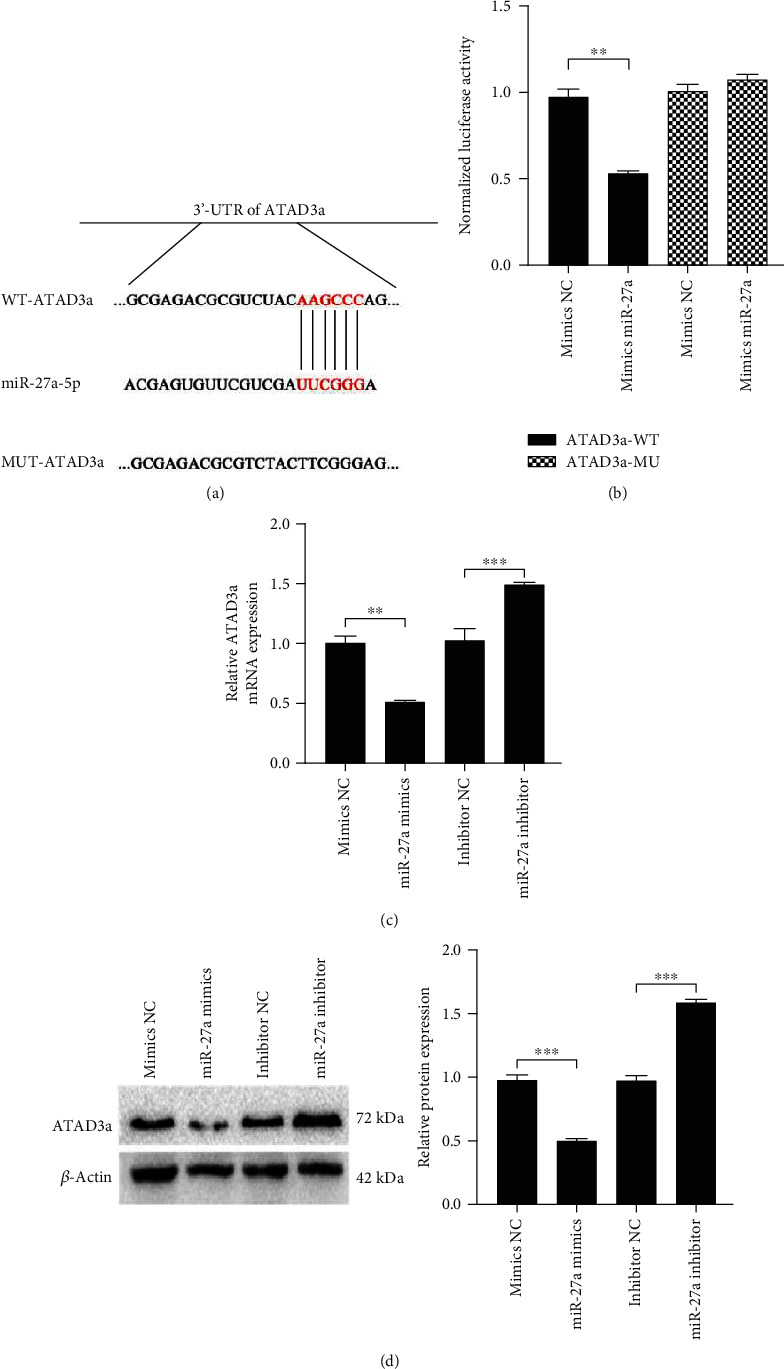
ATAD3a was a target of miR-27a. (a) miR-27a-specific binding on the 3′-UTR of ATAD3a. (b) The relative luciferase activity was analyzed after cotransfecting the pmirGLO luciferase reporter vectors containing wild-type or mutant binding sites with the miR-27a mimic and mimic negative control (NC). (c, d) The mRNA and protein levels of ATAD3a in H9c2 cells transfected with the miR-27a mimics, miR-27a inhibitor, mimics NC, and inhibitor NC were detected by RT-qPCR and Western blotting. All data were obtained from four independent replicate experiments. ^∗∗^*p* < 0.01; ^∗∗∗^*p* < 0.001.

As predicted with TargetScan, miR-27a may bind to the 3′-UTR of ATAD3a (Figure 4(a)), and a luciferase reporter assay was performed to verify the specific interaction of these two molecules. A decrease in the relative luciferase activity was observed when ATAD3a-WT was cotransfected with the miR-27a mimic compared with when ATAD3a-WT was cotransfected with the mimic NC. In contrast, no significant alteration was observed when ATAD3a-MUT was cotransfected with the miR-27a mimic (Figure 4(b)). Furthermore, overexpression of miR-27a reduced the mRNA and protein levels of ATAD3a, while suppression of miR-27a enhanced the mRNA and protein levels of ATAD3a (Figures 4(c) and 4(d)). Taken together, these results suggested that ATAD3a is a target of miR-27a.

### 4.5. miR-27a Regulated the Translocation of AIF from the Mitochondria to the Nucleus

**Figure 5:**
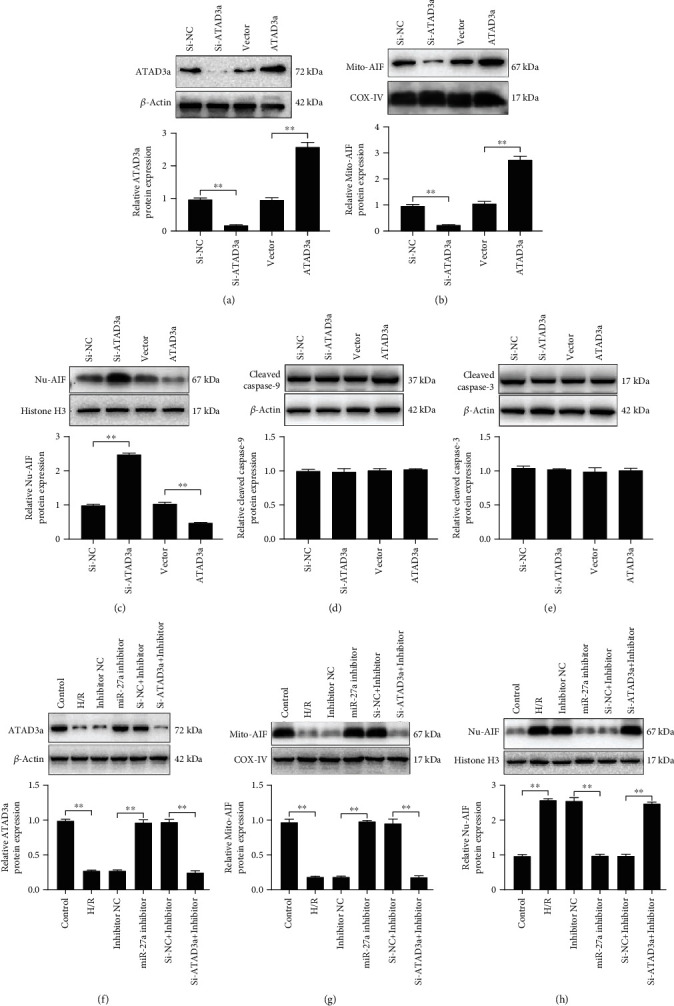
miR-27a regulated the translocation of apoptosis-inducing factor (AIF) from the mitochondria to the nucleus. Small interfering RNA targeting ATAD3a (Si-ATAD3) and matched negative control (Si-NC) ATAD3a plasmid and matched empty vector were transfected into H9c2 cells. The level of ATAD3a (A) and the level of AIF in the mitochondria (b), the nuclei (c), cleaved caspase-9 (d) and cleaved caspase-3 (E) were detected by Western blotting. H9c2 cells were transfected with the miR-27a inhibitor or inhibitor NC, together with the si-ATAD3a or si-NC, and the cells were then exposed to 8 h of hypoxia and 3 h of reoxygenation. Alterations in the levels of ATAD3a (f), AIF in the mitochondria (g), and AIF in the nucleus (h) were detected by Western blotting. All data were obtained from four independent replicate experiments. ^∗∗^*p* < 0.01.

AAA-domain-containing 3A (ATAD3a) is recognized as an antiapoptotic factor [24, 25], but the mechanism underlying its antiapoptotic effect is not well understood. It was found that suppression of ATAD3A led to an increase in the AIF levels in the nucleus and a decrease in the AIF levels in the mitochondria, and overexpression of ATAD3A led to the opposite effects (Figures 5(a)–5(c)). However, modulation of ATAD3a expression did not alter the levels of cleaved caspase-9 and cleaved caspase-3 (Figures 5(d) and 5(e)). These results suggested that ATAD3a regulated apoptosis by modulating the translocation of AIF from the mitochondria to the nucleus, and this apoptosis occurred in a caspase-independent manner. It was further discovered that inhibition of miR-27a expression suppressed the H/R-induced translocation of AIF from the mitochondria to the nucleus, and this effect was reversed by knockdown of ATAD3a (Figures 5(f)–5(h)), demonstrating that miR-27a regulated the translocation of AIF from the mitochondria to the nucleus via the modulation of ATAD3a.

### 4.6. The Effect of miR-27a on H/R-Induced Myocardial Injury Was Mediated by ATAD3a

**Figure 6:**
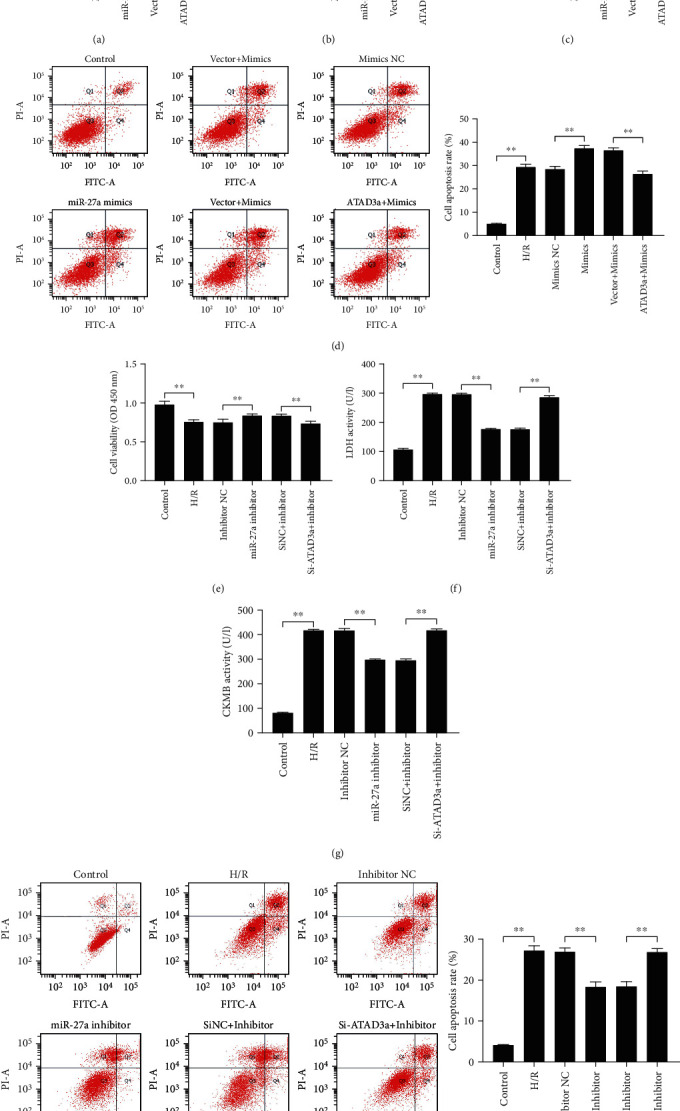
The effect of miR-27a on hypoxia/reoxygenation- (H/R-) induced myocardial injury was mediated by ATAD3a. H9c2 cells were transfected with the miR-27a mimic or mimic negative control (NC), together with the ATAD3a plasmid or empty vector, and then, the cells were subjected to 8 h of hypoxia and 3 h of reoxygenation. (a) Analysis of cell viability by CCK-8 assay. (b, c) Measurement of LDH and CK-MB activity in the culture medium. (d) Cell apoptosis was detected by flow cytometry. H9c2 cells were transfected with the miR-27a inhibitor or inhibitor NC, together with the small interfering RNA targeting ATAD3a (Si-ATAD3) and matched negative control (Si-NC), and the cells were subjected to 8 h of hypoxia and 3 h of reoxygenation. (e) Analysis of cell viability by CCK-8 assay. (f, g) Measurement of LDH and CK-MB activity in the culture medium. (h) Cell apoptosis was detected by flow cytometry. All data were obtained from four independent replicate experiments. ^∗∗^*p* < 0.01.

To investigate the regulatory role of ATAD3a in the effect of miR-27a on H/R-induced myocardial injury, we examined whether the effect of miR-27a on MIRI was compromised by downregulation or upregulation of ATAD3a expression in vitro. As shown in Figures 6(a)–6(c), the decrease in cell viability, increase in cardiac enzyme secretion, and increase in apoptosis after treatment with the miR-27a mimic were compromised when the cells were cotransfected with ATAD3a-overexpressing plasmids. In contrast, the beneficial effects of miR-27a inhibitor treatment on the increase in cell viability, decrease in cardiac enzyme secretion, and decrease in apoptosis were abrogated when the cells were cotransfected with si-ATAD3a (Figures 6(d)–6(f)). Altogether, these results demonstrated that the effect of miR-27a on H/R-induced myocardial injury was mediated by ATAD3a.

### 4.7. Inhibition of miR-27a Using AAV9-Mediated Gene Therapy Mitigated MIRI Ex Vivo

**Figure 7:**
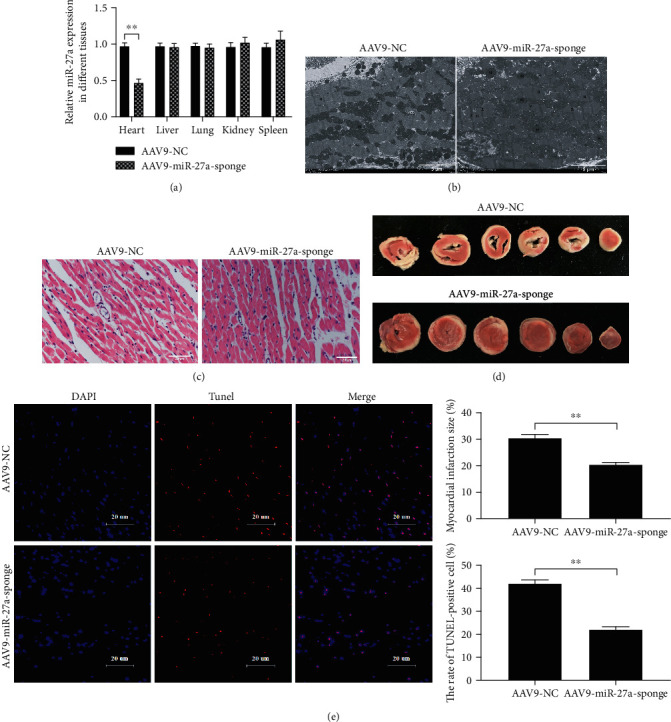
Inhibition of miR-27a using AAV9-mediated gene therapy mitigated MIRI ex vivo. (a) Selective inhibition of miR-27a in the myocardium in vivo was achieved by injecting AAV9-miR-27a-sponge through the rat tail vein. The rat injected with AAV9-miR-27a-negative control (NC) acts as a control. The isolated rat hearts were subjected to 30 min of ischemia, followed by 90 min of reperfusion. Myocardial structure damage was detected by (b) transmission electron microscopy and (c) HE staining, *n* = 3. (d) Myocardial infarct size was measured by TTC staining, *n* = 6. (e) Apoptosis in the myocardium was detected by TUNEL staining, *n* = 6. ^∗∗^*p* < 0.01.

We also examined whether inhibition of miR-27a expression using AAV9-mediated gene therapy could ameliorate MIRI in an isolated rat heart model. After injection of rat hearts with the AAV9-rno-miR-27a sponge, the level of miR-27a in the rat hearts was notably decreased (Figure 7(a)). In addition, the rat hearts treated with the AAV9-miR-27a sponge exhibited less structural damage in the mitochondria and myocardial fibers (Figures 7(b) and 7(c)), decreased myocardial infarct size (Figure 7(d)), and decreased apoptosis rates (Figure 7(e)) compared with those injected with NC. Taken together, these results suggested that inhibition of miR-27a using AAV9-mediated gene therapy mitigated MIRI ex vivo.

## 5. Discussion

The expression of miR-27a in myocardial ischemic disease remains controversial. Several studies have shown that the level of miR-27a is increased in the peripheral blood mononuclear cells (PBMCs) of CAD patients [34, 35]. However, Xue et al. [36] found that the levels of circulating miR-27a were not significantly different in the peripheral blood of AMI patients compared with control subjects. In the present study, miR-27a expression was markedly induced in myocardia exposed to I/R and cardiomyocytes treated with H/R, which is consistent with the findings of Liu JY et al. in mice [19]. However, miR-27a expression was notably downregulated in a simple model of myocardial hypoxia without reoxygenation [37]. Therefore, we speculated that the discrepancy in the above findings may be ascribed to the differences in the models.

Several studies have demonstrated that transcription factors can regulate miR-27a expression by binding to the miR-27a promoter [38, 39]. For instance, mutant p53 (p53-273H) suppresses miR-27a expression by specifically binding to the miR-27a promoter region (nts -2899 to -2675) and subsequently enhances EGF-mediated ERK1/2 activation in breast and lung cancer cells [38]. Additionally, HIF-1*α*, a key transcription factor related to the regulation of gene expression under hypoxic conditions, leads to an increase in miR-27a expression by directly binding to the promoter region of miR-27a [39]. In our study, we found that the upregulation of c-Fos expression in myocardia exposed to I/R was positively correlated with the expression of miR-27a. Furthermore, it was found that c-Fos could directly bind to the promoter region of miR-27a and positively regulate miR-27a expression in vitro. Thus, we suggested that c-Fos, whose expression is induced during MIRI, transcriptionally activated miR-27a, which partly accounted for the upregulation of miR-27a during MIRI.

ATAD3a was verified as a novel target of miR-27a in our study. Although ATAD3a is reported to act as an antiapoptotic factor in lung adenocarcinoma [24] and prostate cancer [25], the detailed underlying mechanism is not known. In the present study, we found that ATAD3a could regulate the translocation of AIF from the mitochondria to the nucleus, which is consistent with the findings of Chiang et al. [40]. After AIF enters the nucleus, DNA fragmentation is triggered, resulting in the initiation of apoptosis [41]. However, modulation of ATAD3a expression has little effect on the activation of caspases, which demonstrated that the regulation of apoptosis by ATAD3a occurs in a caspase-independent manner. It has been documented that miR-27a plays an important role in the regulation of apoptosis [42–44]. miR-27a can regulate cell apoptosis by targeting Fas-associated protein with death domain (FADD) [42], SMAD5 [43], PPAR gamma [44], etc. In this study, it was demonstrated that the translocation of AIF from the mitochondria to the nucleus and the apoptosis induced by H/R were regulated by miR-27a, and these effects were dependent on ATAD3a, which provided new insight into the mechanism by which miR-27a contributes to the regulation of I/R-induced myocardial apoptosis.

Increasing evidence has shown that miR-27a can be used as a target for gene therapy [45–47]. An antisense oligonucleotide specific for miR-27a (antagomiR-27a) and miR-27a sponges are two common methods used to inhibit the function of miR-27a [48, 49]. Ge et al. [45] suggested that antagomiR-27a inhibits glioblastoma cell growth in vitro and in vivo. Additionally, antagomiR-27a has a good therapeutic effect on the prevention of diabetic nephropathy [46]. In addition, Salah et al. [47] found that miR-27a sponges could inhibit the invasion and metastasis of osteosarcoma. A recent study demonstrated that miR-27a mediates the protective effect of high thoracic epidural block on MIRI [12], but whether inhibition of miR-27a expression decreases the direct protective effect on MIRI was not determined. In the present study, MIRI was alleviated in isolated rat hearts by treatment with an AAV-9 vector expressing miR-27a sponges, which suggested that miR-27a could be used as a target for gene therapy in MIRI in the future.

The MIRI model generated by the Langendorff approach in this study is a robust model for studying ischemia-reperfusion injury [50]. In the present study, Langendorff-perfused rat hearts without I/R that stained by TTC did not indicate any infarct size, while the hearts induced by 30 min ischemia and 90 min reperfusion showed large infarct areas, which provides a direct evidence for MIRI. However, we must acknowledge that there is also a limitation in this model. Because the isolated rat heart is deprived of neurohumoral control, this lack of systemic influence should be further evaluated [51]. Despite of this limitation, the data generated by the Langendorff model still provides useful information in understanding the role of the c-Fos/miR-27a/ATAD3a axis in MIRI.

## 6. Conclusions

Our study demonstrated that c-Fos functions as an upstream regulator of miR-27a and that miR-27a regulates the translocation of AIF from the mitochondria to the nucleus by targeting ATAD3a, thereby contributing to MIRI. These findings provide new insight into the role of the c-Fos/miR-27a/ATAD3a axis in MIRI and suggest that miR-27a could be used as a target for gene therapy in MIRI.

## Data Availability

The data used to support the findings of this study are available from the corresponding author upon request.
